# The Role of Case Syncretism in Agreement Attraction: A Comprehension Study

**DOI:** 10.3389/fpsyg.2022.829112

**Published:** 2022-05-06

**Authors:** Natalia Slioussar, Varvara Magomedova, Polina Makarova

**Affiliations:** ^1^School of Linguistics, National Research University Higher School of Economics, Moscow, Russia; ^2^Faculty of Liberal Arts and Sciences, Saint Petersburg State University, Saint Petersburg, Russia; ^3^Institute for Cognitive Studies, Saint Petersburg State University, Saint Petersburg, Russia

**Keywords:** gender agreement, attraction, comprehension, syncretism, Russian

## Abstract

Many production and comprehension experiments have studied attraction errors in agreement, primarily in number (e.g., “The key to the cabinets were rusty”). Studies on gender agreement attraction are still sparse, especially in comprehension. We present two self-paced reading experiments on Russian focusing on the role of syncretism in this phenomenon. Russian nouns are inflected for case and number, and some forms have the same inflections (are syncretic). In several experiments on Slovak, it was shown that both head and attractor syncretism play a role for gender agreement in production. We demonstrate for the first time that this is also the case in comprehension. The role of head noun syncretism has not been analyzed in any previous comprehension studies, also for number agreement. We conclude that syncretic forms create uncertainty, which is crucial for agreement disruption. These results are better compatible with retrieval approaches to agreement attraction. We discuss the implications of our findings for the nature of the retrieval cues used to establish morphosyntactic dependencies. The question whether case marking modulates agreement attraction in comprehension has also been addressed in a study on Armenian, and it found no evidence of such influence. We offer an explanation of the conflicting findings from several studies based on the syntactic constructions they used as materials.

## Introduction

The phenomenon of agreement attraction is analyzed in many production and comprehension studies. They found that errors like (1a) are produced more often and are more easily missed in comprehension than errors like (1b)—presumably, because in (1a) the dependent noun phrase (NP) *the cabinets* (termed *attractor*) interferes with the agreement between the verb and the head of the subject NP. In particular, comprehension experiments showed that processing attraction errors like (1a) is associated with smaller reading time delays, fewer grammaticality judgment errors, and smaller P600 amplitudes than processing other agreement errors like (1b).

(1) a. *The key to the cabinets were rusty.
      b. *The key to the cabinet were rusty.

Attraction effects were observed both in number and in gender agreement across a variety of languages (e.g., Bock and Miller, 1991; Vigliocco et al., 1995, 1996; Clifton et al., 1999; Pearlmutter et al., 1999; Vigliocco and Franck, 1999; Franck et al., 2002, 2006, 2008; Hartsuiker et al., 2003; Solomon and Pearlmutter, 2004; Eberhard et al., 2005; Badecker and Kuminiak, 2007; Staub, 2009, 2010; Wagers et al., 2009; Dillon et al., 2013; Tanner et al., 2014; Slioussar and Malko, 2016; Slioussar, 2018; Hammerly et al., 2019; Tucker et al., 2021). The phenomenon attracts researchers’ attention because agreement is one of the basic grammatical operations that we strive to understand and because attraction effects allowed studying many other important questions. However, the underlying mechanisms of attraction and the role of different syntactic, semantic, and morphological factors that influence it are still under debate.

In this study, we present two comprehension experiments on Russian that aim to clarify the role of syncretism in agreement attraction. In the languages with morphological case marking, word forms in different cases may coincide, which makes them morphologically ambiguous, or syncretic. To give an example, accusative plural forms of many Russian nouns are syncretic with nominative plural ones (e.g., *stoly* “table_NOM.PL = ACC.PL_”), while dative plural forms are never morphologically ambiguous (e.g., *stolam* “table_DAT.PL_”).

As we show in more detail below, syncretism was found to affect agreement attraction in several previous production and comprehension studies on different languages (e.g., Hartsuiker et al., 2003; Badecker and Kuminiak, 2007; Slioussar, 2018). However, Avetisyan et al. (2020) did not detect any influence of case marking on number agreement attraction in their comprehension study on Armenian. They drew a general conclusion that different retrieval cues, like case and number, are used differentially when establishing syntactic dependencies.

We continue exploring this question and seek to explain *prima facie* conflicting findings to shed new light on the nature of retrieval mechanisms. Firstly, we demonstrate that both attractor and head syncretism are crucial for agreement attraction in comprehension—while attractor syncretism was addressed in several studies, only Badecker and Kuminiak (2007) assessed the role of head syncretism, but only in production. Secondly, our study is the first to analyze any syncretism effects in gender agreement processing. Thirdly, we hypothesize that the differences between Avetisyan et al. (2020) and other studies, including ours, may be due to the different syntactic constructions used as materials. As a result, we argue that retrieval cues are used in combination at least in certain syntactic constructions.

The paper has the following structure. In the next section, we briefly present some information on Russian grammar that is necessary to understand our experimental designs. Then, we discuss two major theoretical approaches to attraction phenomena and several previous studies that are most relevant for our project: the ones that focus on the role of syncretism and case marking and on gender agreement attraction in comprehension. After that, we turn to the present study.

### Nominal Paradigms and Gender Agreement in Russian

In Russian, nouns belong to one of three genders: masculine (M), feminine (F), or neuter (N). Gender agreement can be observed only in singular: on adjectives, participles, and past tense verb forms (plural forms are the same for all three genders). Russian nouns are inflected for two numbers and six cases: nominative, genitive, dative, accusative, instrumental, and locative (also called as prepositional). The choice of inflection depends on the inflectional class and subclass the noun belongs to.

There are different approaches to the system of inflectional noun classes, or declensions, in Russian. We will rely on the most widely accepted one (e.g., Shvedova, 1980; Aronoff, 1994; Halle, 1994), in which nouns are divided into three declensions. The first declension (D1) contains the majority of feminine nouns and a small number of masculine nouns. The majority of masculine nouns and all neuter nouns belong to the second declension (D2). The third declension (D3) contains a small group of feminine nouns. Table 1 provides some examples (only in singular, because plural forms will not be discussed in this paper).

**Table 1:** Singular paradigms of the nouns *stena* “wall_F_,” *pol* “floor_M_,” *kot* “cat_M_,” *okno* “window_N_,” and *dver’* “door_F_.”

	D1	D2			D3
	F/M	M inanimate	M animate	N	F
Nominative	*stena*	*pol*	*kot*	*okno*	*dver’*
Genitive	*steny*	*pola*	*kota*	*okna*	*dveri*
Dative	*stene*	*polu*	*kotu*	*oknu*	*dveri*
Accusative	*stenu*	*pol*	*kota*	*okno*	*dver’*
Instrumental	*stenoj*	*polom*	*kotom*	*oknom*	*dver’ju*
Locative	*stene*	*pole*	*kote*	*okne*	*dveri*

Table 1 does not show different inflectional subclasses that depend on the final consonant of the stem (velar, affricate, other non-palatalized, and palatalized). However, this variation does not affect syncretism patterns that we are primarily interested in this paper.*’* stands for the letter *soft sign* that usually indicates that the preceding consonant is palatalized but has a number of other functions.

Now let us discuss syncretism patterns paying special attention to nominative forms because they are crucial for attraction effects. In the first declension, nominative forms are morphologically unambiguous (only locative and dative singular are syncretic). In the second declension, accusative singular coincides with nominative singular in neuter and inanimate masculine nouns. In animate masculine nouns, accusative and genitive singular are syncretic. In the third declension, there is a lot of syncretism, and nominative and accusative singular always coincide. Therefore, in our experimental designs, we will choose nouns from different declensions depending on whether syncretic or non-syncretic forms are required.

### Previous Studies of Agreement Attraction

Different models that seek to explain attraction can be divided into two major groups. The first one assumes that the syntactic representation of the subject NP may be faulty or ambiguous (e.g., Franck et al., 2002; Eberhard et al., 2005; Staub, 2009, 2010). Some versions of this approach, like the Marking and Morphing model (Eberhard et al., 2005; Hammerly et al., 2019), are readily applicable only to number agreement. The others can be used to account for gender agreement as well: they claim that when we construct the subject NP in production or in comprehension, we may determine its gender feature incorrectly because of the dependent noun interference. The second approach postulates an access error, which is made when the predicate needs to find the agreement controller (Badecker and Kuminiak, 2007; Wagers et al., 2009; Dillon et al., 2013). As we show in more detail below, the effects of syncretism are better explained within this approach.

The role of syncretism for agreement attraction was first tested on the German language (Hartsuiker et al., 2003). German inflects nouns, adjectives, articles, and pronouns into four cases: nominative, genitive, dative, and accusative. In plural, many nouns have the same form in all cases, but the case is visible on the definite article, which has the forms *die, der, den,* and *die*, respectively. Thus, the form *die* is ambiguous between nominative and accusative plural.^1^ Hartsuiker et al. (2003) demonstrated that this plays a role for number agreement attraction in production: the number of errors with syncretic attractors, as in (2a), was significantly higher than with non-syncretic ones, as in (2b).

(2) a. *die Stellungnahme gegen die Demonstrationen*^2^
        the_NOM.SG_ position against the_ACC.PL(=NOM.PL)_ demonstrations
      b. *die Stellungnahme zu den Demonstrationen*
        the_NOM.SG_ position on the_DAT.PL(≠NOM.PL)_ demonstrations

Badecker and Kuminiak (2007) worked on Slovak. The system of declensions in Slovak is similar to the one in Russian that was described in the previous section. Badecker and Kuminiak confirmed Hartsuiker et al.’s (2003) generalization in a production study of gender agreement and extended it in an important way. They showed that significant attraction effects are observed only when *both* the dependent noun (a potential attractor) and the head noun are syncretic, as in (3). In other words, for the subject-verb agreement to be disrupted, not only the attractor should look like a nominative subject, but also the form of the subject should not unambiguously point to the nominative case.

(3) *pohár na mlieko*
      glass_NOM.SG(=ACC.SG)_ for milk_ACC.SG(=NOM.SG)_

Badecker and Kuminiak (2007) note that their results can be more readily explained by retrieval models, rather than representational ones. According to the versions of the representational approach that are compatible with gender agreement attraction, the subject NP may be erroneously marked with the features of the dependent noun rather than the head. It is not clear why this should depend on the syncretism of the dependent noun and especially of the head.

Slioussar (2018) demonstrated that attractor syncretism plays a role not only in production, but also in comprehension in a study of number agreement in Russian. Her experiments explored the distinction between systematic and accidental syncretism that receives different treatments in different morphological theories (e.g., Zwicky, 1991; Blevins, 1995; Stump, 2001; Bobaljik, 2002; Baerman et al., 2005; Müller, 2011). In particular, the syncretism of nominative and accusative forms, as in (4b), is regarded as an example of the former, whereas the syncretism of nominative plural and genitive singular forms, as in (4c), is considered an example of the latter (e.g., McCreight and Chvany, 1991; Müller, 2004; Wiese, 2004; Wunderlich, 2004; Baerman et al., 2005).

(4) a. *ssylka na dokument*
        reference_NOM.SG_ to article_ACC.SG(≠NOM.PL)_
      b. *ssylka na dokumenty*
        reference_NOM.SG_ to article_ACC.PL(=NOM.PL)_
      c. *material dlja stat’i*
        material_NOM.SG_ for article_GEN.SG(=NOM.PL)_
      d. *material dlja statej*
        material_NOM.SG_ for article_GEN.PL(≠NOM.PL)_

Slioussar (2018) found that both in production and in comprehension, attraction effects were the most pronounced with the subject NPs like (4b). Then came the NPs like (4c), in which the dependent noun was not plural, but its form was syncretic with nominative plural. For the subject NPs like (4d) with non-syncretic dependent nouns, no evidence of attraction could be detected.

Like Badecker and Kuminiak (2007) and Slioussar (2018) argued that her results supported the retrieval approach. When we produce a verb form or process it in comprehension, we should retrieve the agreement controller (maybe, in comprehension this happens only when the form does not match our expectations based on the features of the subject NP we have just processed—in this case, rechecking is initiated). Slioussar concluded that we are looking for a form that has not only the relevant number features, but also the nominative feature, i.e., compound retrieval cues are used. Moreover, the feature sets activated due to syncretism also play a role, although systematic syncretism is more effective in disrupting agreement than accidental syncretism. However, Slioussar did not control for the syncretism of head nouns in her materials.

Avetisyan et al. (2020) explored the role of case marking in number agreement attraction in a series of comprehension experiments on Armenian. In all studies mentioned above, potential attractors were dependent nouns in a subject NP. Avetisyan et al. chose relative clauses—another construction widely used in attraction research. An English example with an attraction error and without it is given in (5).

(5) The hypotheses one entertains/*entertain influence the outcome.

Armenian is a pro-drop SOV language, so a sentence can start both with a nominative noun, as in (6a), or with an accusative one, as in (6b), if the subject was dropped. Avetisyan et al. (2020) compared examples like (6a) and (6b) and found significant attraction effects in both conditions. Moreover, there was no evidence that accusative case marking on the head of the relative clause attenuated these effects compared to the nominative condition.

(6) a. nkaričnerë, oronč k’andakagorçë arhamarhec’/*arhamarhec’in
        painter_NOM.PL.DEF_ that_ACC.PL_ sculptor_NOM.SG.DEF_ ignore_AOR.3SG_/ignore_AOR.3PL_
      b. nkaričnerin, oronč k’andakagorçë arhamarhec’/*arhamarhec’in
        painter_ACC.PL.DEF_ that_ACC.PL_ sculptor_NOM.SG.DEF_ ignore_AOR.3SG_/ignore_AOR.3PL_

Thus, Avetisyan et al. (2020) did not manipulate syncretism in their study, but their results are nevertheless highly relevant. They concluded that accusative attractors are retrieved as effectively as nominative ones and, more generally, that case and number are not used as compound retrieval cues. Explaining why Slioussar (2018) found a different pattern in Russian, they noted that case ambiguous experimental items were lexically different from the case unambiguous ones in her study and suggested that “case differences may have been confounded with semantic differences between the nouns in the subject phrase” (Avetisyan et al., 2020, p. 3).

Creating syncretic and non-syncretic conditions with the same lexical items is indeed impossible. Slioussar (2018) used prepositions that require accusative or genitive case: even if she tried to build her examples using the same nouns, the semantic relations between them determined by the preposition would be very different. Badecker and Kuminiak (2007) selected nouns with different paradigms, with or without nominative-accusative syncretism. Although this may introduce some additional noise in the data, it is not immediately clear how this could influence attraction. Larger attraction effects were found with subject NPs in which the head and the dependent noun are more closely connected semantically. It would be a surprising coincidence if semantic connections consistently happened to be closer in the syncretic conditions, but this cannot be categorically excluded. However, if case marking played no role, Slioussar (2018) would have found larger attraction effects with non-syncretic plural dependents like (4d) than with syncretic singular ones like (4c). A significant difference in the opposite direction, which was not confounded by using different lexical items, can only be explained by the influence of case marking.

Given these *prima facie* conflicting findings, we come back to the role of syncretism in agreement attraction in the present study but focus on gender agreement. No previous study addressed the role of syncretism for gender agreement attraction in comprehension. Moreover, there are in general very few comprehension studies focusing on gender agreement attraction—mainly on Spanish, but also on Russian, Arabic, and Greek (Acuña-Fariña et al., 2014; Martin et al., 2014; Slioussar and Malko, 2016; Paspali and Marinis, 2020; Villata and Franck, 2020; Alonso et al., 2021; Tucker et al., 2021).

Spanish does not inflect nouns for case, so the problem of syncretism is irrelevant. The Russian and Greek studies (Slioussar and Malko, 2016; Paspali and Marinis, 2020) did not test this factor as. Only syncretic attractors were used as the authors assumed that this would increase attraction effects, allowing them to study other factors of interest. The syncretism of heads was not controlled for. In case of Slioussar and Malko (2016), this might have happened because they started by replicating in Russian the first production experiment by Badecker and Kuminiak (2007) that also did not take head syncretism into account. Finally, Tucker et al. (2021) studied relative clauses in Arabic, not manipulating the case factor.

Two findings by Slioussar and Malko (2016) are important for the present study. Firstly, they discovered that in comprehension, gender agreement attraction can be observed only with feminine and neuter heads, but not with masculine ones. Analyzing possible explanations of this pattern would take us too far afield—into an extensive discussion concerning the role of feature markedness for agreement attraction and possible sources of cross-linguistic differences (for example, Tucker et al. (2021) found a different pattern in Arabic). This does not seem to be necessary in this study because our experimental designs take Slioussar and Malko’s (2016) finding into account but do not depend on its explanation. In the two reading experiments we conducted, we used only feminine heads to make sure that attraction was possible and manipulated the syncretism of head and attractor nouns.

Secondly, the head nouns in Slioussar and Malko’s (2016) experiments were always inanimate. This means that all neuter and masculine heads were syncretic. As for feminine heads, they happened to be non-syncretic because the absolute majority of feminine nouns belongs to the first declension with morphologically unambiguous nominative singular forms. Attraction effects were observed in both neuter and feminine head conditions, although, judging by average reading times, they were less pronounced in the latter case. This means that head syncretism might play a role in attraction effects in comprehension, but at least they are not categorically excluded with non-syncretic heads.

### The Present Study

We conducted two moving-window word-by-word self-paced reading experiments on gender agreement processing in Russian. We used stimulus sentences with complex subject NPs, potential attractors were dependent nouns inside these NPs. As we mentioned in the previous section, the role of syncretism has never been tested for gender agreement attraction in comprehension. In Experiment 1, we compared sentences with syncretic and non-syncretic attractors. In addition to that, we manipulated the animacy of the attractor assuming that whether the gender is purely grammatical (on inanimate nouns) or conceptual (on animate nouns) might play a role for gender agreement processing. In Experiment 2, we focused on the head syncretism factor that has never been assessed in comprehension experiments either for gender or for number agreement.

## Experiment 1

In this experiment, we tested whether the syncretism and animacy of the dependent noun play a role for attraction in gender agreement.

### Participants

A 78 native speakers of Russian (32 males and 46 females) aged 18–44 took part in Experiment 1. All participants were naïve to the experimental hypotheses. No participant took part in more than one experiment. All experiments reported in this paper were carried out in accordance with the Declaration of Helsinki and the existing Russian and international regulations concerning ethics in research. All participants provided informed consent.

### Materials

A 48 sets of stimulus sentences in four conditions were constructed for Experiment 1. Examples are given in (7a–d)–(9a–d). All sentences were 8 words long and had the same syntactic structure: N_1_ (head)—preposition—N_2_ (dependent)—copula (*byt’* “to be”)—adjective/participle^3^—three words modifying the predicate. All heads were inanimate feminine nouns from the first declension, so their nominative singular forms were morphologically unambiguous. Predicates were in the past tense, with feminine or masculine agreement (yielding grammatical and ungrammatical conditions). Dependent nouns were feminine or masculine: attraction effects might be expected in the latter cases, while the former served as control conditions. Based on the animacy and syncretism of dependent nouns, stimulus sentences were divided into three groups (16 stimuli in each group): with syncretic inanimate dependent nouns, with non-syncretic inanimate ones, and with non-syncretic animate ones.^4^

(7) Syncretic inanimate dependent group:
      a. *FF: Nagruzka na otrasl’ byla snižena posle otmeny naloga*
        burden_F.NOM.SG_ on industry_F.ACC.SG(=NOM.SG)_ was_F.SG_ reduced_F.SG_ after canceling_GEN.SG_ tax_GEN.SG_
      b. FM: *Nagruzka na otrasl’ byl snižen posle otmeny naloga
        burden_F.NOM.SG_ on industry_F.ACC.SG(=NOM.SG)_ was_M.SG_ reduced_M.SG_ after canceling_GEN.SG_ tax_GEN.SG_
      c. *MF: Nagruzka na sektor byla snižena posle otmeny naloga*
        burden_F.NOM.SG_ on sector_M.ACC.SG(=NOM.SG)_ was_F.SG_ reduced_F.SG_ after canceling_GEN.SG_ tax_GEN.SG_
      d. MM: *Nagruzka na sektor byl snižen posle otmeny naloga
        burden_F.NOM.SG_ on sector_M.ACC.SG(=NOM.SG)_ was_M.SG_ reduced_M.SG_ after canceling_GEN.SG_ tax_GEN.SG_

“The burden on the industry/sector was reduced after the cancellation of the tax.”

(8) Non-syncretic inanimate dependent group:
      a. *FF: Vyboina v plitke byla zadelana posle smeny podryadčika*
        pothole_F.NOM.SG_ in tile_F.LOC.SG_ was_F.SG_ repaired_F.SG_ after change_GEN.SG_ contractor_GEN.SG_
      b. FM: *Vyboina v plitke byl zadelan posle smeny podryadčika
        pothole_F.NOM.SG_ in tile_F.LOC.SG_ was_M.SG_ repaired_M.SG_ after change_GEN.SG_ contractor_GEN.SG_
      c. *MF: Vyboina v asfal’te byla zadelana posle smeny podryadčika*
        pothole_F.NOM.SG_ in asphalt_M.LOC.SG_ was_F.SG_ repaired_F.SG_ after change_GEN.SG_ contractor_GEN.SG_
      d. MM: *Vyboina v asfal’te byl zadelan posle smeny podryadčika
        pothole_F.NOM.SG_ in asphalt_M.LOC.SG_ was_M.SG_ repaired_M.SG_ after change_GEN.SG_ contractor_GEN.SG_

“The pothole in the tile/asphalt was repaired after the change of the contractor.”

(9) Non-syncretic animate dependent group:
      a. *FF: Perepiska s podrugoj byla prervana na prodolžitelniy srok*
        correspondence_F.NOM.SG_ with girlfriend_F.INS.SG_ was_F.SG_ interrupted_F.SG_ for long period_ACC.SG_
      b. FM: *Perepiska s podrugoj byl prervan na prodolžitelniy srok
        correspondence_F.NOM.SG_ with girlfriend_F.INS.SG_ was_M.SG_ interrupted_M.SG_ for long period_ACC.SG_
      c. *MF: Perepiska s priyatelem byla prervana na prodolžitelniy srok*
        correspondence_F.NOM.SG_ with friend_M.INS.SG_ was_F.SG_ interrupted_F.SG_ for long period_ACC.SG_
      d. MM: *Perepiska s priyatelem byl prervan na prodolžitelniy srok.
        correspondence_F.NOM.SG_ with friend_M.INS.SG_ was_M.SG_ interrupted_F.SG_ for long period_ACC.SG_

“The correspondence with a girlfriend/friend was suspended for an extended period.”

We can speak of attraction in the cases, in which ungrammatical masculine predicates cause significantly smaller reading time delays in the masculine dependent condition than in the feminine dependent condition. The delays are measured in comparison with the corresponding grammatical conditions with feminine predicates, so balancing feminine and masculine dependent nouns were not required. Nevertheless, we closely matched their average length, frequency, and the natural logarithm of the frequency in every stimulus group to facilitate comparisons across conditions by making stimuli more homogenous.

As for the expected differences between the three stimulus groups, we made the following hypotheses. If attraction is possible only with syncretic dependent nouns, we would observe it only in the inanimate syncretic group. If some attraction effects can also be observed with non-syncretic attractors, the animacy factor could be tested. Gender features are interpretable on nouns denoting humans, while on inanimate nouns, they are semantically empty.^5^ Some previous findings show that gender features are more salient in the former case, so we wanted to test whether this could affect attraction.^6^ Of course, it would be optimal to have the fourth group of stimuli with syncretic animate dependent nouns, but this is impossible in Russian: in all masculine animate nouns, nominative forms are non-syncretic.

Sentences in different conditions were distributed across four experimental lists. As a result, every list contained 48 stimulus sentences (in each of the three groups, there were four sentences in the FF, FM, MF, and MM conditions) and 96 grammatically correct filler sentences. Some filler sentences were structurally similar to stimuli (although the head of the subject NP was not feminine, to create some diversity), the others were not. Filler sentences were 6–9 words long. Every list started with 10 filler sentences, after which point target and filler sentences were pseudo-randomized (with at most two target sentences with errors in a row).

### Procedure

The sentences were presented on a PC using the Ibex Farm platform.^7^ We used moving-window word-by-word self-paced reading methodology. Each trial began with a sentence in which all words were masked with dashes while spaces remained intact. Participants were pressing the space bar to reveal a word and re-mask the previous one. Word-by-word reading times were recorded.

One-third of the sentences was followed by forced choice comprehension questions to ensure that the participants were reading properly. Two answer variants were presented one above the other. Participants pressed “1” to choose the answer above, and “2” to choose the answer below. Participants were instructed to read at a natural pace and answer questions as accurately as possible. They were not informed in advance that some sentences would contain errors. An experimental session lasted around 13 min.

### Analysis

We analyzed participants’ question-answering accuracy and reading times. On average, participants answered only 5.3% of questions incorrectly (14.8% at most). Given the low number of mistakes, a breakdown of RTs into correct and incorrect question trials was not performed. Reading times that exceeded a threshold of 2.5 standard deviations, by region and condition, were excluded (Ratcliff, 1993). In total, 2.1% of the data was excluded (at most 4.2% per region and condition).

We modeled the data with mixed-effects regressions in *R* software (www.r-project.org) using the *lmer* function from the *lme4* package (Bates et al., 2015). To obtain *p* values from the *t* values given by the model, we used the *lmerTest* package (Kuznetsova et al., 2015). Random intercepts and random slopes by participant and by item were included in the model. In different analyses reported below, the gender of the dependent noun, the gender of the verb (i.e., grammaticality), and the syncretism of the dependent noun were treated as fixed effects. For the predictors, we used treatment contrast coding. Feminine on the dependent noun was coded as 0, masculine (mismatched with the head) as 1. Feminine on the verb (grammatical) was coded as 0, masculine (ungrammatical) as 1. Syncretic forms were coded as 1, non-syncretic as 0. Animate nouns were coded as 1, inanimate as 0.

### Results and Discussion

**Figure 1:**
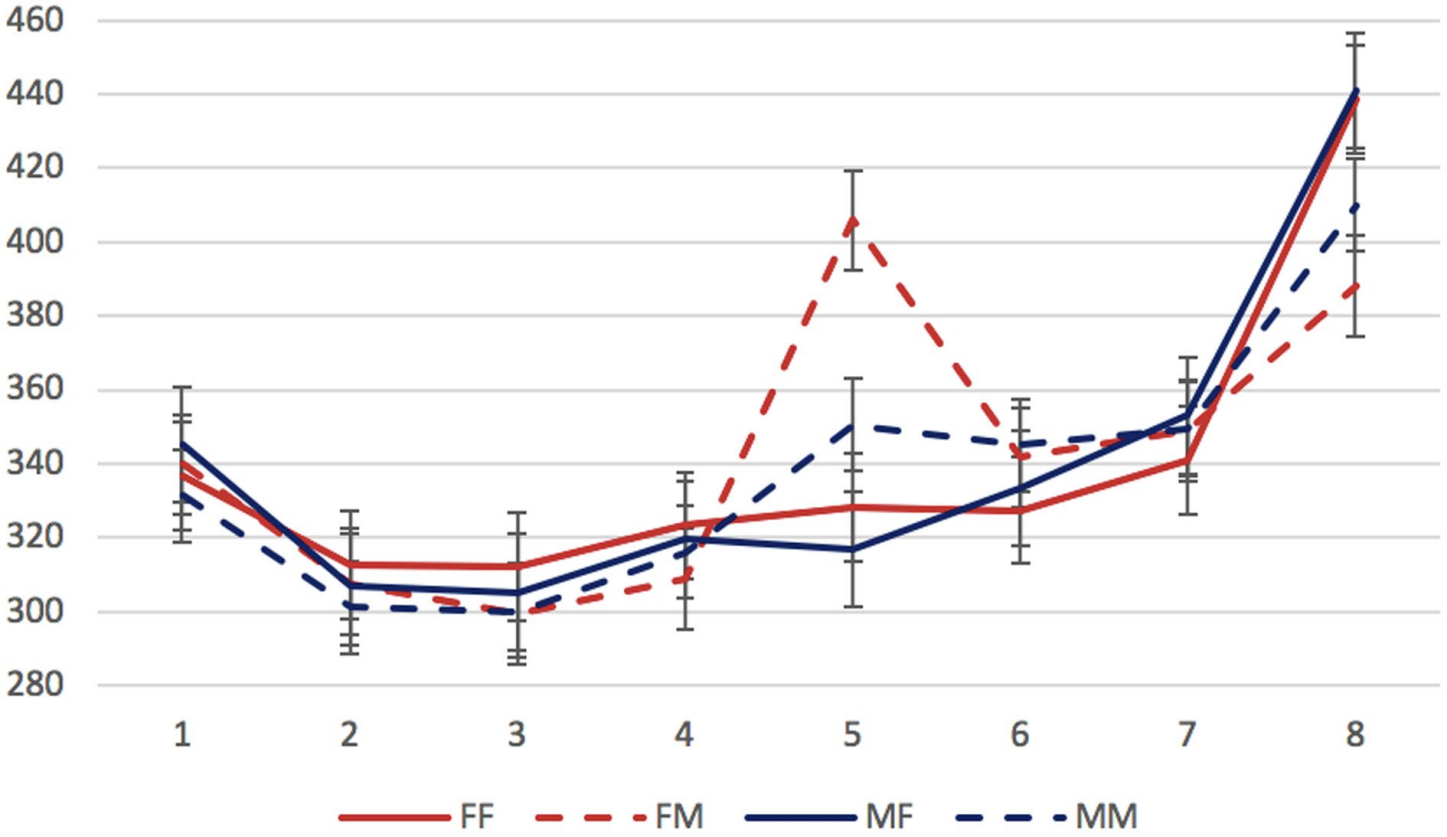
Experiment 1, the syncretic inanimate dependent group: mean RTs per region (in ms) in the four experimental conditions. Regions: N_1_ (head)—preposition—N_2_ (dependent)—copula (*byt’* “to be”)—adjective/participle—three words modifying the predicate. Error bars represent the standard error of the condition mean.

**Figure 2:**
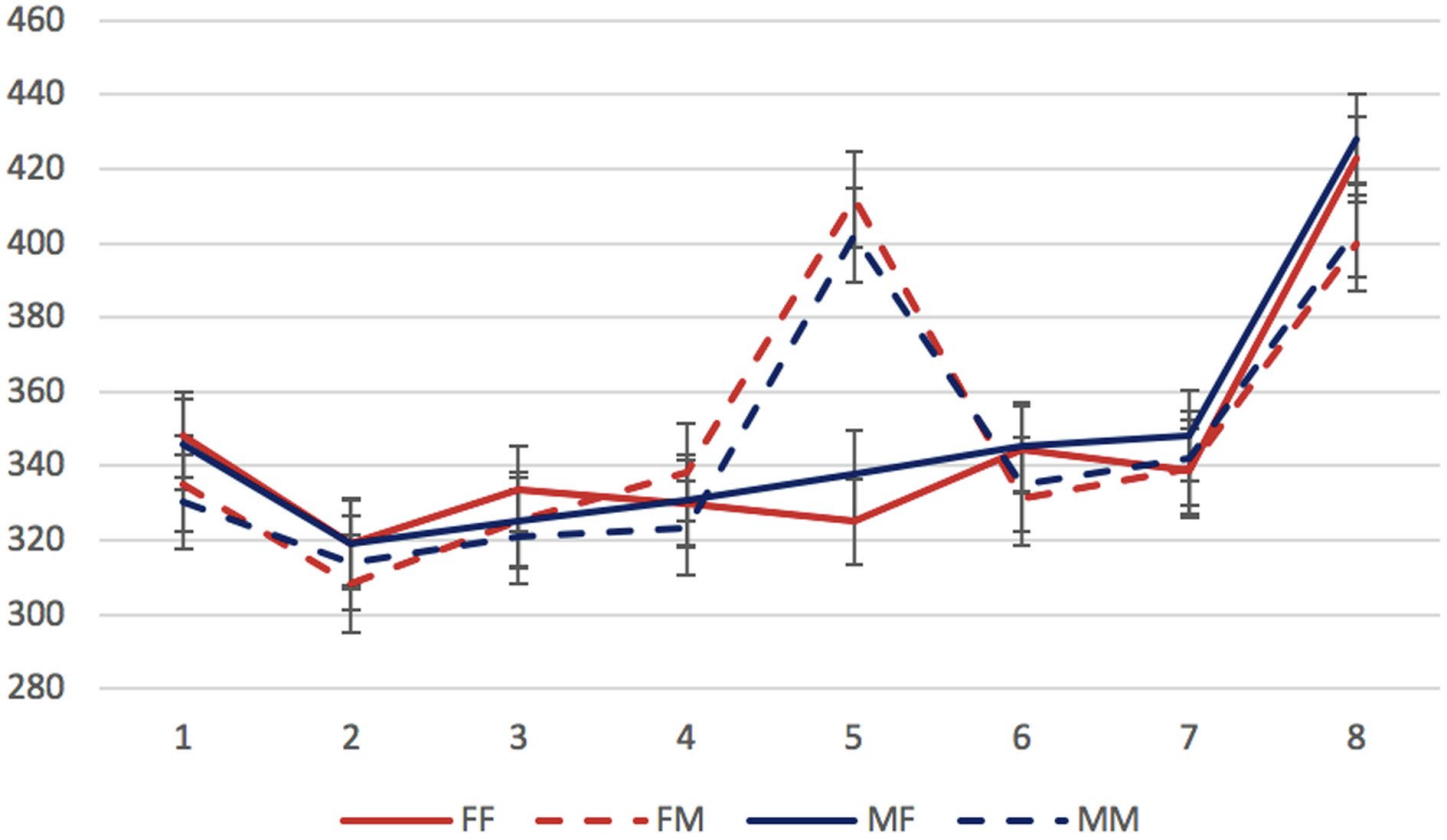
Experiment 1, the non-syncretic inanimate dependent group: mean RTs per region (in ms) in the four experimental conditions. Regions: N_1_ (head)—preposition—N_2_ (dependent)—copula (*byt’* “to be”)—adjective/participle—three words modifying the predicate. Error bars represent the standard error of the condition mean.

**Figure 3:**
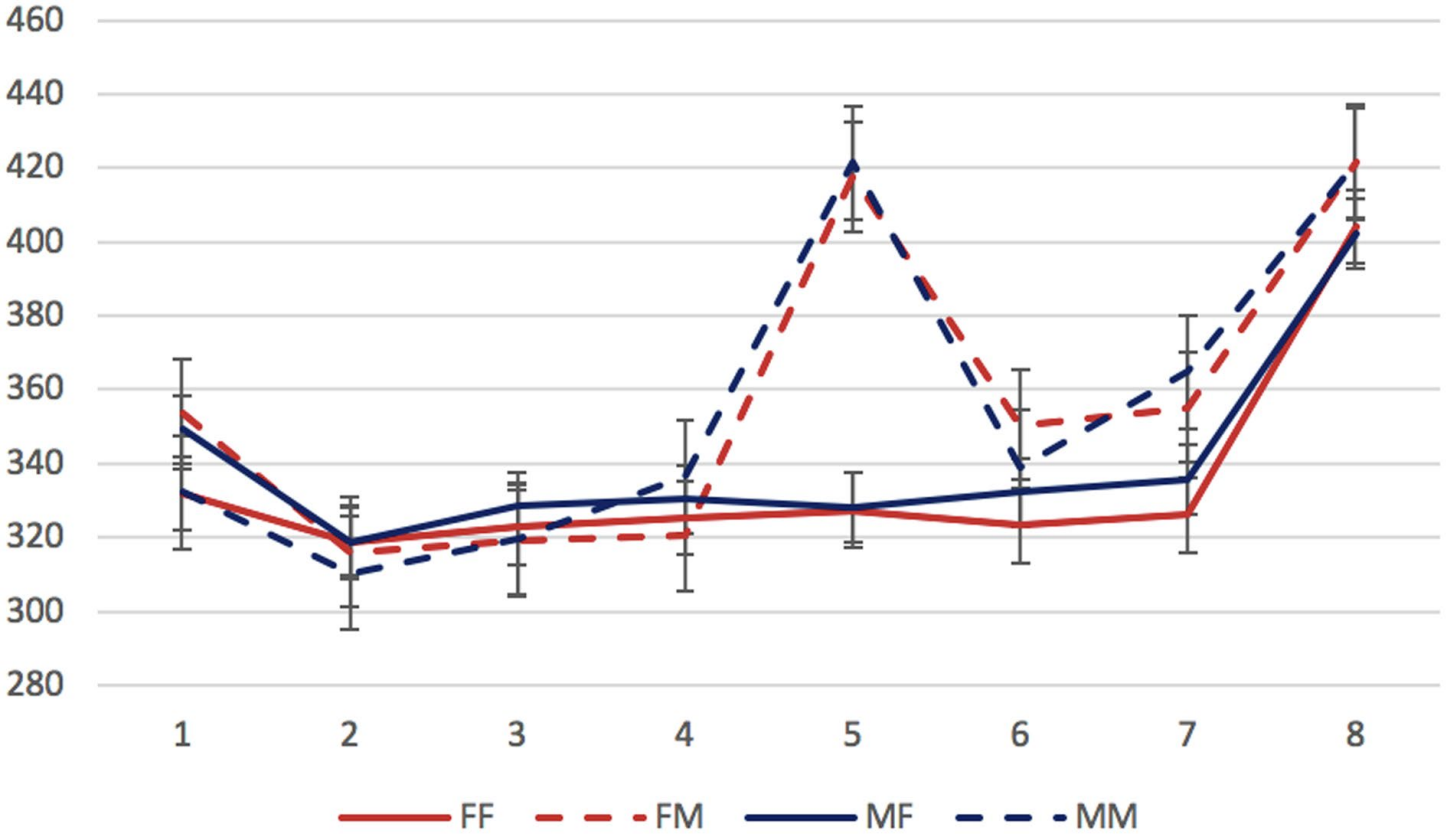
Experiment 1, the non-syncretic animate dependent group: mean RTs per region (in ms) in the four experimental conditions. Regions: N_1_ (head)—preposition—N_2_ (dependent)—copula (*byt’* “to be”)—adjective/participle—three words modifying the predicate. Error bars represent the standard error of the condition mean.

Mean reading times per region in different conditions are presented in Figures 1–3.

We started by analyzing data from the three stimulus groups separately. The factors of interest were the gender of the dependent noun and of the predicate (grammaticality). In all three groups, significant differences were found only in the region 5 containing an adjective or participle. Mean reading times for this region in all experimental conditions are presented in Table 2.

**Table 2:** Experiment 1: mean RTs (in ms) and standard deviations (in parentheses) in region 5 in different experimental conditions.

	FF	FM	MF	MM
Syncretic inanimate dependent group	328 (102)	406 (111)	317 (159)	350 (118)
Non-syncretic inanimate dependent group	325 (126)	412 (152)	338 (139)	402 (142)
Non-syncretic animate dependent group	327 (109)	418 (159)	328 (123)	422 (136)

In both non-syncretic groups, only the grammaticality factor was significant (*β* = 64.90, *SE* = 7.91, *t* = 8.21, *p* < 0.01 for the inanimate dependent group; *β* = 89.10, *SE* = 11.05, *t* = 8.06, *p* < 0.01 for the animate dependent group). In the syncretic group, not only the grammaticality factor, but also the interaction of grammaticality and dependent noun gender reached significance (*β* = 34.18, *SE* = 7.07, *t* = 4.83, *p* < 0.01; *β* = −44.76, *SE* = 10.03, *t* = −4.46, *p* < 0.01, respectively). This means that only in this group, attraction effects could be detected: only in the sentences with syncretic dependent nouns, error-related reading time delays were significantly smaller when the gender of these nouns matched the gender of the ungrammatical predicate.

To assess the contribution of syncretism directly, we selected sentences with masculine attractors from the two inanimate groups. The factors of interest were attractor syncretism and grammaticality. This comparison revealed the significance of the grammaticality factor (*β* = 66.57, *SE* = 7.83, *t* = 8.51, *p* < 0.01) and of the interaction between grammaticality and syncretism (*β* = −31.73, *SE* = 11.03, *t* = −2.88, *p* < 0.01). As for the animacy factor, since both non-syncretic groups showed no evidence of attraction, its role for attraction could not be assessed.

## Experiment 2

In this experiment, we tested whether the syncretism of the head noun plays a role for attraction in gender agreement.

### Participants

82 native speakers of Russian (36 males and 46 females) aged 18–49 took part in Experiment 2.

### Materials

A 32 sets of stimulus sentences in four conditions were constructed for Experiment 2. Examples are given in (10a–d)–(11a–d). Like in Experiment 1, all sentences were 8 words long and had the same syntactic structure: N_1_ (head)—preposition—N_2_ (dependent)—copula (*byt’* “to be”)—adjective/participle—three words modifying the predicate. All heads were inanimate feminine nouns, and based on their declension, the stimuli were divided into two groups: with first declension heads that have a morphologically unambiguous nominative singular form and with third declension heads in a syncretic form (16 stimuli in each group). Dependent nouns could be feminine or masculine and were always in a syncretic accusative singular form (Experiment 1 showed that otherwise, no attraction would be observed). Predicates were in the past tense, with feminine or masculine agreement (grammatical and ungrammatical conditions).

(10) Syncretic head group:
      a. *FF: Rec’ pro moral’ byla skučnoj s pervyx slov*
        speech_F.NOM.SG(=ACC.SG)_ about moral_F.ACC.SG(=NOM.SG)_ was_F.SG_ boring_F.SG_ from first words_GEN.PL_
      b. FM: *Rec’ pro moral’ byl skučnym s pervyx slov
        speech_F.NOM.SG(=ACC.SG)_ about moral_F.ACC.SG(=NOM.SG)_ was_M.SG_ boring_M.SG_ from first words_GEN.PL_
      c. *MF: Rec’ pro etiket byla skučnoj s pervyx slov*
        speech_F.NOM.SG(=ACC.SG)_ about etiquette_M.ACC.SG(=NOM.SG)_ was_F.SG_ boring_F.SG_ from first words_GEN.PL_
      d. MM: *Rec’ pro etiket byl skučnym s pervyx slov
        speech_F.NOM.SG(=ACC.SG)_ about etiquette_M.ACC.SG(=NOM.SG)_ was_M.SG_ boring_M.SG_ from first words_GEN.PL_

“The speech about morality/etiquette was boring from the very first few words.”

(11) Non-syncretic head group:
      a. *FF: Ocenka za četvert’ byla vysokoj u priležnogo učenika*
        grade_F.NOM.SG_ for term_F.ACC.SG(=NOM.SG)_ was_F.SG_ high_F.SG_ at diligent student_GEN.SG_
      b. FM: *Ocenka za četvert’ byl vysokim u priležnogo učenika
        grade_F.NOM.SG_ for term_F.ACC.SG(=NOM.SG)_ was_M.SG_ high_M.SG_ at diligent student_GEN.SG_
      c. *MF: Ocenka za semestr byla vysokoj u priležnogo učenika*
        grade_F.NOM.SG_ for semester_M.ACC.SG(=NOM.SG)_ was_F.SG_ high_F.SG_ at diligent student_GEN.SG_
      d. MM: *Ocenka za semestr byl vysokim u priležnogo učenika
        grade_F.NOM.SG_ for semester_M.ACC.SG(=NOM.SG)_ was_M.SG_ high_M.SG_ at diligent student_GEN.SG_

“The diligent student got an excellent mark for the term/semester.”

In this experiment, only one factor is tested, so the hypothesis is very simple. Badecker and Kuminiak (2007) found that head syncretism significantly increased attraction effects in their production study of gender agreement in Slovak. If head syncretism also plays a role for agreement attraction in comprehension, more pronounced effects (i.e., smaller reading time delays in the MM condition compared to the FM condition) are expected in the syncretic head group than in the non-syncretic head group.

Sentences in different conditions were distributed across four experimental lists. Every list contained 32 stimulus sentences and 68 grammatically correct filler sentences. Like in Experiment 1, fillers could be structurally similar to stimuli or not. They were 6–9 words long. Every list started with four filler sentences, after which point target and filler sentences were pseudo-randomized (with at most two target sentences with errors in a row).

### Procedure

The procedure was the same as in the Experiment 1. An experimental session lasted around 10 min.

### Analysis

We analyzed participants’ question-answering accuracy and reading times. On average, participants answered only 6.2% of questions incorrectly (15.1% at most). Given the low number of mistakes, a breakdown of RTs into correct and incorrect question trials was not performed. Reading times that exceeded a threshold of 2.5 standard deviations, by region and condition, were excluded (Ratcliff, 1993). For one participation, this led to the exclusion of more than 15% responses, so we did not include his data in further analysis. After removing this participant, 1.8% of the data was excluded (at most 3.4% per region and condition).

The statistical analysis was the same as in the Experiment 1. In different analyses reported below, the gender of the dependent noun, the gender of the verb (i.e., grammaticality), and the syncretism of the head noun were treated as fixed effects. For the predictors, we used treatment contrast coding. Feminine on the dependent noun was coded as 0, masculine (mismatched with the head) as 1. Feminine on the verb (grammatical) was coded as 0, masculine (ungrammatical) as 1. Syncretic forms were coded as 1, non-syncretic as 0.

### Results and Discussion

**Figure 4:**
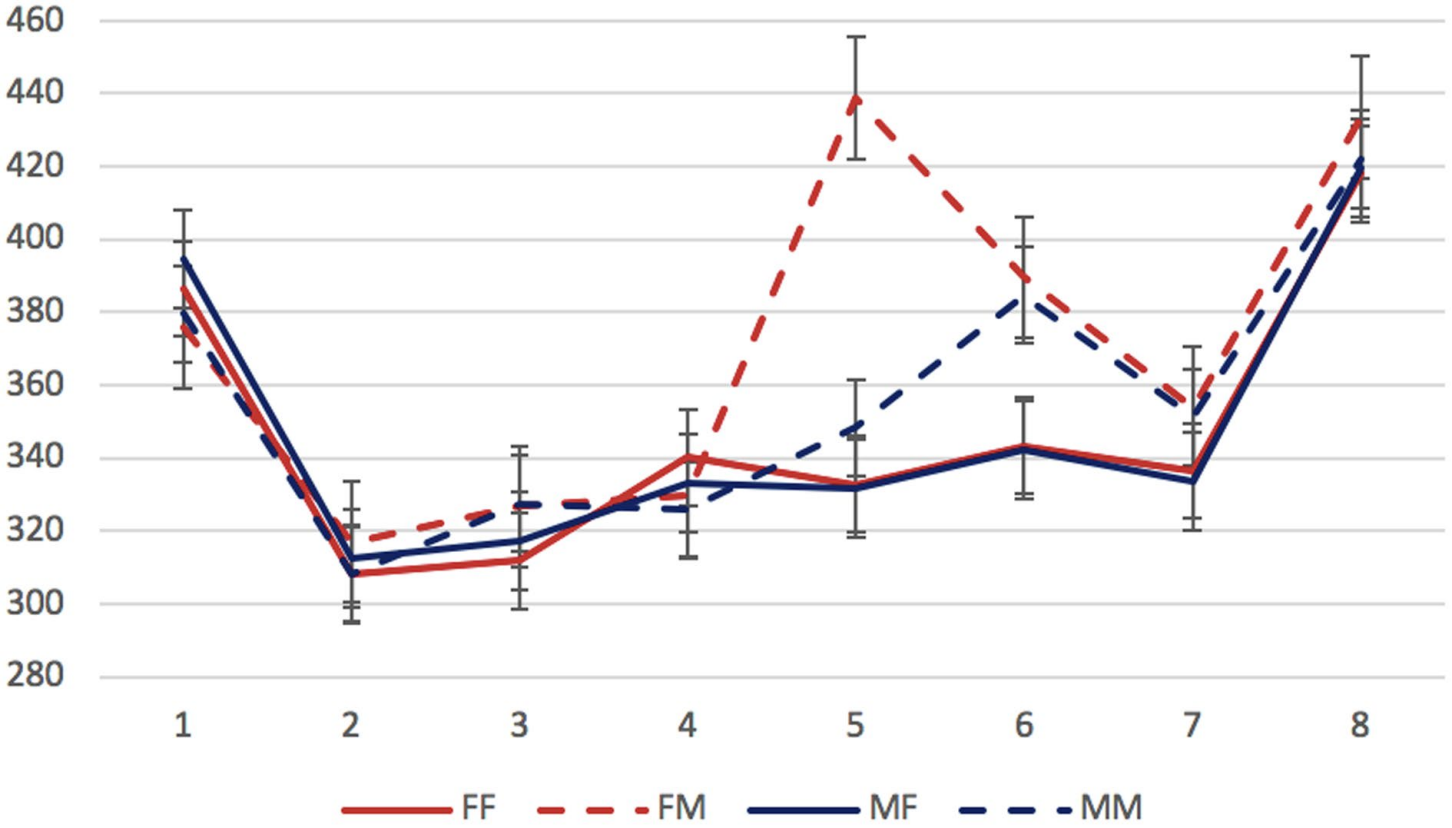
Experiment 2, the syncretic head group: mean RTs per region (in ms) in the four experimental conditions. Regions: N_1_ (head)—preposition—N_2_ (dependent)—copula (*byt’* “to be”)—adjective/participle—three words modifying the predicate. Error bars represent the standard error of the condition mean.

**Figure 5:**
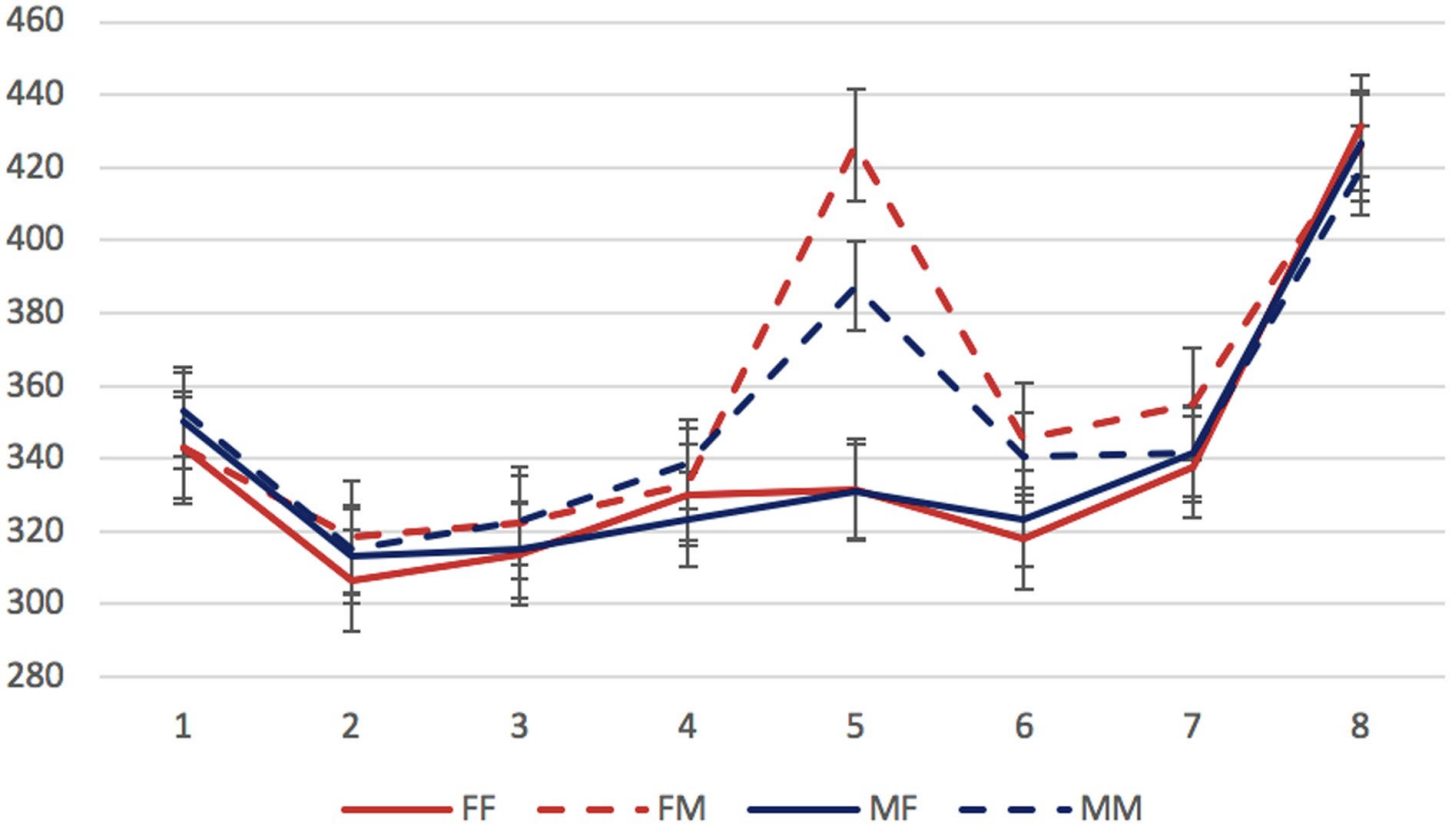
Experiment 2, the non-syncretic head group: mean RTs per region (in ms) in the four experimental conditions. Regions: N_1_ (head)—preposition—N_2_ (dependent)—copula (*byt’ “*to be”)—adjective/participle—three words modifying the predicate. Error bars represent the standard error of the condition mean.

Mean reading times per region in different conditions are presented in Figures 4, 5.

Like in the Experiment 1, we firstly analyzed data from the two stimulus groups separately. The factors of interest were the gender of the dependent noun and of the predicate (grammaticality). Significant differences were found in the regions 5 and 6 containing an adjective or participle and the first word from the constituent modifying the predicate. Mean reading times for these regions in all experimental conditions are presented in Table 3.

**Table 3:** Experiment 2: mean RTs (in ms) and standard deviations (in parentheses) in regions 5 and 6 in different experimental conditions.

	Region 5				Region 6			
	FF	FM	MF	MM	FF	FM	MF	MM
Syncretic head group	333 (115)	439 (198)	332 (116)	348 (155)	343 (101)	390 (131)	342 (104)	385 (115)
Non-syncretic head group	332 (99)	426 (123)	331 (97)	387 (120)	318 (94)	346 (116)	323 (95)	340 (109)

In the region 5, both the grammaticality factor and the interaction between grammaticality and dependent noun gender reached significance in both groups (*β* = 106.79, *SE* = 9.01, *t* = 11.86, *p* < 0.01; *β* = −85.24, *SE* = 12.77, *t* = −6.68, *p* < 0.01 for the syncretic group; *β* = 94.36, *SE* = 6.22, *t* = 15.16, *p* < 0.01; *β* = −36.83, *SE* = 8.78, *t* = −4.19, *p* < 0.01 for the non-syncretic group). In other words, attraction effects can be observed in both groups. In the region 6, only the grammaticality factor was significant in both groups (*β* = 27.73, *SE* = 5.72, *t* = 4.85, *p* < 0.01 for the syncretic group; *β* = 46.95, *SE* = 6.44, *t* = 7.29, *p* < 0.01 for the non-syncretic head group).

To assess the contribution of head syncretism directly, we selected sentences with masculine attractors from the two groups. The factors of interest were head syncretism and grammaticality. This comparison revealed the significance of the grammaticality factor (*β* = 57.55, *SE* = 7.35, *t* = 7.83, *p* < 0.01) and of the interaction between grammaticality and syncretism (*β* = −37.30, *SE* = 10.43, *t* = −3.58, *p* < 0.01). In other words, we can conclude that attraction can be observed both with syncretic and with non-syncretic heads, but head syncretism significantly influences the size of the effect.

## General Discussion

We demonstrated that both head syncretism and attractor syncretism are important for attraction effects in comprehension. These questions have not been explored before for gender agreement, while for number agreement, only attractor syncretism has been analyzed in a previous comprehension study on Russian (Slioussar, 2018). Badecker and Kuminiak (2007) argue based on their production data on gender agreement in Slovak that both factors are prerequisites. Our results suggest a different picture for comprehension: attraction is possible with non-syncretic heads and syncretic dependent nouns, but the syncretism of the head significantly enhances the effect. With non-syncretic dependent nouns, no evidence of attraction was found, which is consistent with Slioussar’s (2018) results for number agreement.

Our conclusions are corroborated by previous findings by Slioussar and Malko (2016). They found attraction effects both with neuter heads, which are always syncretic in nominative singular, and with feminine heads, which happened to be non-syncretic in their experiments because most Russian feminine nouns belong to the first declension with morphologically unambiguous nominative singular forms. Judging by average reading times, attraction effects were much more pronounced with neuter heads. Slioussar and Malko did not estimate this difference statistically and did not comment on it, but our findings reveal its possible source.

However, our conclusions contradict the ones reached by Avetisyan et al. (2020). As we mentioned in the introduction, only Slioussar (2018) and the present study assessed the role of case syncretism for agreement attraction in comprehension. But Avetisyan et al. (2020) chose a different way to approach the case factor. In their comprehension study on Armenian, they used stimuli with relative clauses (RCs), as in (12), and found equally significant attraction effects with nominative and accusative RC heads. They concluded that case marking does not affect agreement attraction in comprehension and that number and case retrieval cues are used separately.

(12) nkaričnerë/nkaričnerin, oronč k’andakagorçë arhamarhec’ / *arhamarhec’in
     painter_NOM.PL.DEF_/painter_ACC.PL.DEF_ that_ACC.PL_ sculptor_NOM.SG.DEF_ ignore_AOR.3SG_/ignore_AOR.3PL_

Trying to reconcile conflicting findings, we focused on the syntactic structure of (12). The object of the verb “ignored” in (12) is not the noun “painters,” which is outside of the relative clause, but *oronč “*that_ACC.PL_.” We believe that *oronč,* and not the RC head is the attractor. Obviously, *oronč* and the RC head are closely connected,^8^ in particular, *oronč* refers to the painters and copies the number feature of the RC head—but not its case feature. The RC head may appear in different cases depending on the matrix predicate, but *oronč* is always unambiguously marked with accusative. We hypothesize that this is crucial for attraction, and this is why Avetisyan et al. (2020) did not find any difference between conditions with nominative and accusative RC heads.

If our hypothesis is on the right track, it may lead us to a more general question. Why do we observe significant attraction effects with *oronč*, but not with unambiguously non-nominative dependent nouns in Slioussar (2018) or in the present study? We can offer a very tentative answer and outline directions for further research that would be necessary to test it. Attraction effects have been detected not only with complex subject NPs and with relative clauses—direct and indirect objects were found to disrupt agreement between the subject and the verb in many studies on different languages. None of these studies manipulated the case syncretism factor. But we can note that in several experiments, object attractors were unambiguously non-nominative (e.g., Franck et al., 2006, 2010, 2020), and nevertheless, significant effects were observed.

It may be the case that direct objects (including object clitics and the elements like *oronč*) are more efficient attractors than dependent nouns inside the subject NP. From the syntactic point of view, this would not be surprising because in many languages, verbs agree with direct objects, but never with the nouns embedded in the subject NPs. Therefore, case syncretism may be less important for the former than for the latter.

To test this hypothesis properly, one would have to manipulate the case factor comparing different attractor types in a single experiment on one case-marking language. Leaving this for further research, we can draw intermediate conclusions based on the previous studies and on the present one. The role of case marking may be different for different attractor types, but at least for dependent nouns inside a subject NP, case syncretism plays a crucial role for agreement attraction both in production and in comprehension.

The role of syncretism is more readily compatible with retrieval approaches to agreement attraction, rather than with representational ones. According to the versions of the representational approach that can in principle be applied to gender agreement attraction, the subject NP may be erroneously marked with the features of the dependent noun rather than the head. As Badecker and Kuminiak (2007) noted discussing their production data, it is not clear why the incidence of such errors should depend on the case syncretism of the dependent noun and especially of the head.

Slioussar (2018) who was the first to study the role of attractor syncretism in comprehension concluded that during retrieval, the system looks for a combination of features, trying to find a form that has the relevant number or gender feature *and* the nominative case feature. If the dependent noun is in the form syncretic with nominative, it may be erroneously retrieved, creating attraction effects. Otherwise, no evidence of attraction can be found.

We adopt this approach to account for the role of attractor syncretism in our experiments. Contra Avetisyan et al. (2020), we conclude that not only case and number, but also case and gender retrieval cues can be used in combination. However, this approach cannot explain the role of head syncretism. The fact that accusative attractors are syncretic with nominative becomes relevant because the system searches for a nominative feature. Why is the fact that nominative heads are syncretic with accusative attractors relevant if the system does not search for an accusative feature?

Apparently, syncretism has a more general impact on processing. A morphologically ambiguous form creates uncertainty activating two feature sets and therefore makes the retrieval less automatic and gives an opportunity for the attractor to be retrieved.

To understand the nature of this uncertainty, let us keep in mind that Russian has flexible word order and not only a subject, but also an object may be sentence-initial. Of course, there is a strong preference to interpret sentence-initial NPs as subjects (Sekerina, 1997). Moreover, the predicates in our stimuli consisted to the “to be” verb in the past tense and an adjective or participle. No such predicate is compatible with an accusative object in Russian. However, since case syncretism may create syntactic ambiguity in other contexts, it is associated with higher uncertainty.^9^

Finally, let us turn to another problem that is discussed in many comprehension studies of agreement attraction. Representational approaches to attraction predict so-called *ungrammaticality illusions* in comprehension, while retrieval approaches are compatible both with their presence and their absence. Attraction effects in ungrammatical sentences (when reading time delays associated with an agreement error are diminished if a number of gender feature on the dependent noun matches the feature of the ungrammatical predicate form) can be described as *grammaticality illusions*. Several authors have argued that *ungrammaticality illusions* are also possible (e.g., Nicol et al., 1997; Pearlmutter et al., 1999): correct predicate forms are processed more slowly in the sentences in which the features of the dependent noun are mismatched with the features of the head. These authors hypothesized that the subject NP may have an incorrect representation both in grammatical and in ungrammatical sentences, which would create wrong expectations about the predicate form and produce ungrammaticality illusions in the former and grammaticality illusions in the latter.

The debate on ungrammaticality illusions on number agreement attraction data was plagued by the fact that attraction is found only with singular heads and plural dependents, but not in the opposite situation. Plural forms take longer to process for independent reasons. Wagers et al. (2009) argued that if these effects are controlled for, we can still observe grammaticality illusions, but not ungrammaticality ones, i.e., the latter are epiphenomenal. Nevertheless, there still remained some room for controversy. Our data may be instrumental here. We found no evidence of ungrammaticality illusions, and our target sentences had feminine heads and feminine or masculine dependent nouns. There are no reasons to expect feminine forms to be processed faster (if anything, the opposite could be true because masculine gender is much more frequent in Russian).

## Data Availability Statement

The raw data supporting the conclusions of this article will be made available by the authors, without undue reservation.

## Ethics Statement

Ethical review and approval was not required for the study on human participants in accordance with the local legislation and institutional requirements. The patients/participants provided their written informed consent to participate in this study.

## Author Contributions

NS planned the experiments and supervised the work, designed the first experiment, and took part in interpreting results from both experiments. VM did the statistical analysis of the data and took part in interpreting the results. PM created materials for the second experiment and collected the data. All authors contributed to the article and approved the submitted version.

## Funding

The work on this project was carried out in the framework of the Basic Research Program at the National Research University Higher School of Economics, Russia.

## Conflict of Interest

The authors declare that the research was conducted in the absence of any commercial or financial relationships that could be construed as a potential conflict of interest.

## Publisher’s Note

All claims expressed in this article are solely those of the authors and do not necessarily represent those of their affiliated organizations, or those of the publisher, the editors and the reviewers. Any product that may be evaluated in this article, or claim that may be made by its manufacturer, is not guaranteed or endorsed by the publisher.

## Footnotes

^1^All these forms also coincide with some forms in singular, but this does not play a role for the study under discussion.

^2^The preposition *gegen* requires accusative, so the syncretism of the article does not lead to syntactic ambiguity: we definitely know that *die Demonstrationen* is in accusative plural. This is indicated by the glosses, which also show that this form is syncretic with nominative plural, while the dative plural form in (2b) is not.

^3^Russian does not have productive passive voice. Some verbs take the intransitivizing affix-*sja* to convey passive meanings, while the combinations of the “to be” verb and a participle used in our experiments are not analytic verb forms, like in English, but a copular construction.

^4^In the examples below, we marked the gender and syncretism with nominative singular only for head and dependent nouns in the subject NPs because this is relevant for our study. For all other nouns, only number and case are marked. Let us also note that creating *the same* sentences with syncretic and non-syncretic dependent nouns is impossible: all inanimate masculine nouns have syncretic forms in nominative and accusative in Russian, so we used prepositions taking different cases in the syncretic and non-syncretic groups.

^5^For nouns denoting animals, the picture is far from clear (see, e.g., Yanovich, 2012).

^6^In particular, Vigliocco and Franck (1999) observed in a production study on French that participants made significantly fewer gender agreement attraction errors with animate head nouns. Therefore, we decided to test this factor for dependent nouns.

^7^ https://spellout.net/ibexfarm/

^8^How to model such connection syntactically is a matter of debate, but no theory questions its existence.

^9^Notably, it is impossible to explain head syncretism effects simply assuming that syncretic subjects were sometimes interpreted as objects, as least initially. If this were the case, *all* agreement errors, both with feminine and with masculine attractors, would trigger smaller reading time delays than the same errors in the non-syncretic head conditions. This is not what we found.

## References

[ref1] Acuña-Fariña J. C. Meseguer E. Carreiras M. (2014). Gender and number agreement in comprehension in Spanish. Lingua 143, 108–128. doi: 10.1016/j.lingua.2014.01.013

[ref2] Alonso J. G. Cunnings I. Fujita H. Miller D. Rothman J. (2021). Gender attraction in sentence comprehension. Glossa J. General Ling. 20, 1–26.

[ref3] Aronoff M. (1994). Morphology by Itself: Stems and Inflectional Classes. Cambridge, MA: MIT Press.

[ref4] Avetisyan S. Lago S. Vasishth S. (2020). Does case marking affect agreement attraction in comprehension? J. Mem. Lang. 112:104087. doi: 10.1016/j.jml.2020.104087

[ref5] Badecker W. Kuminiak F. (2007). Morphology, agreement and working memory retrieval in sentence production: evidence from gender and case in Slovak. J. Mem. Lang. 56, 65–85. doi: 10.1016/j.jml.2006.08.004

[ref6] Baerman M. Brown D. P. Corbett G. G. (2005). The Syntax-Morphology Interface: A Study of Syncretism. Cambridge: Cambridge University Press.

[ref7] Bates D. Maechler M. Bolker B. Walker S. (2015). lme4: linear mixed-effects models using Eigen and S4. R package version 1.1–8. Available at: http://CRAN.R-project.org/package=lme4 (Accessed October 15, 2021).

[ref8] Blevins J. (1995). Syncretism and paradigmatic opposition. Ling. Philos. 18, 113–152. doi: 10.1007/BF00985214

[ref9] Bobaljik J. (2002). “Syncretism without paradigms: remarks on Williams 1981, 1994,” in Yearbook of Morphology 2001. eds. Booij G. van J. Marle (Dordrecht: Kluwer), 53–85.

[ref10] Bock J. K. Miller C. A. (1991). Broken agreement. Cogn. Psychol. 23, 45–93. doi: 10.1016/0010-0285(91)90003-7 2001615

[ref11] Clifton C. Frazier L. Deevy P. (1999). Feature manipulation in sentence comprehension. Rivista Ling. 11, 11–39.

[ref12] Dillon B. Mishler A. Sloggett S. Phillips C. (2013). Contrasting intrusion profiles for agreement and anaphora: experimental and modeling evidence. J. Mem. Lang. 69, 85–103. doi: 10.1016/j.jml.2013.04.003

[ref13] Eberhard K. Cutting J. C. Bock K. (2005). Making syntax of sense: number agreement in sentence production. Psychol. Rev. 112, 531–559. doi: 10.1037/0033-295X.112.3.531, PMID: 16060750

[ref14] Franck J. Lassi G. Frauenfelder U. H. Rizzi L. (2006). Agreement and movement: A syntactic analysis of attraction. Cognition 101, 173–216. doi: 10.1016/j.cognition.2005.10.003, PMID: 16360139

[ref15] Franck J. Mirdamadi F. Kahnemuyipour A. (2020). Object attraction and the role of structural hierarchy: evidence from Persian. Glossa J. Gen. Ling. 5, 1–17. doi: 10.5334/gjgl.804

[ref16] Franck J. Soare G. Frauenfelder U. H. Rizzi L. (2010). Object interference: The role of intermediate traces of movement. J. Mem. Lang. 62, 166–182. doi: 10.1016/j.jml.2009.11.001

[ref17] Franck J. Vigliocco G. Anton-Mendez I. Collina S. Frauenfelder U. H. (2008). The interplay of syntax and form in sentence production: A cross-linguistic study of form effects on agreement. Lang. Cognit. Processes 23, 329–374. doi: 10.1080/01690960701467993

[ref18] Franck J. Vigliocco G. Nicol J. (2002). Subject–verb agreement errors in French and English: The role of syntactic hierarchy. Lang. Cognit. Processes 17, 371–404. doi: 10.1080/01690960143000254

[ref19] Halle M. (1994). “The Russian declension: An illustration of the theory of distributed morphology,” in Perspectives in Phonology. eds. Cole J. S. Kisseberth C. (Stanford: CSLI Publications), 29–60.

[ref20] Hammerly C. Staub A. Dillon B. (2019). The grammaticality asymmetry in agreement attraction reflects response bias: experimental and modeling evidence. Cogn. Psychol. 110, 70–104. doi: 10.1016/j.cogpsych.2019.01.001, PMID: 30798061

[ref21] Hartsuiker R. J. Schriefers H. J. Bock K. Kikstra G. M. (2003). Morphophonological influences on the construction of subject–verb agreement. Mem. Cogn. 31, 1316–1326. doi: 10.3758/BF03195814, PMID: 15058692

[ref22] Kuznetsova A. Brockhoff P. B. Christensen R. H. B. (2015). lmerTest: tests in linear mixed effects models. R package version 2.0–25. Available at: http://CRAN.R-project.org/package=lmerTest (Accessed October 15, 2021).

[ref23] Martin A. E. Nieuwland M. S. Carreiras M. (2014). Agreement attraction during comprehension of grammatical sentences: ERP evidence from ellipsis. Brain Lang. 135, 42–51. doi: 10.1016/j.bandl.2014.05.001, PMID: 24911918

[ref24] McCreight K. Chvany C. (1991). “Geometric representation of paradigms in a modular theory of grammar,” in Paradigms. The Economy of Inflection. ed. Plank F. (Berlin: Mouton de Gruyter), 91–112.

[ref25] Müller G. (2004). “On decomposing inflection class features: syncretism in Russian noun inflection,” in Explorations in Nominal Inflection. eds. Müller G. Gunkel L. Zifonum G. (Berlin: Mouton De Gruyter), 189–228.

[ref26] Müller G. (2011). Syncretism without under specification: The role of leading forms. Word Struct. 4, 53–103. doi: 10.3366/word.2011.0004

[ref27] Nicol J. Foster K. Veres C. (1997). Subject-verb agreement processes in comprehension. J. Mem. Lang. 36, 569–587. doi: 10.1006/jmla.1996.2497

[ref28] Paspali A. Marinis T. (2020). Gender agreement attraction in Greek comprehension. Front. Psychol. 11, 1–22. doi: 10.3389/fpsyg.2020.00717 32411044 PMC7201047

[ref29] Pearlmutter N. J. Garnsey S. M. Bock K. (1999). Agreement processes in sentence comprehension. J. Mem. Lang. 41, 427–456. doi: 10.1006/jmla.1999.2653

[ref30] Ratcliff R. (1993). Methods for dealing with reaction time outliers. Psychol. Bull. 114, 510–532. doi: 10.1037/0033-2909.114.3.510, PMID: 8272468

[ref31] Sekerina I. A. (1997). The Syntax and Processing of Scrambling Constructions in Russian. Doctoral dissertation. New York: City University of New York.

[ref32] Shvedova N. (1980). Russkaja Grammatika (‘Russian Grammar’). Moscow: Nauka.

[ref33] Slioussar N. (2018). Forms and features: the role of syncretism in number agreement attraction. J. Mem. Lang. 101, 51–63. doi: 10.1016/j.jml.2018.03.006

[ref34] Slioussar N. Malko A. (2016). Gender agreement attraction in Russian: production and comprehension evidence. Front. Psychol. 7:1651. doi: 10.3389/fpsyg.2016.01651, PMID: 27867365 PMC5095607

[ref35] Solomon E. S. Pearlmutter N. J. (2004). Semantic integration and syntactic planning in language production. Cogn. Psychol. 49, 1–46. doi: 10.1016/j.cogpsych.2003.10.001, PMID: 15193971

[ref36] Staub A. (2009). On the interpretation of the number attraction effect: response time evidence. J. Mem. Lang. 60, 308–327. doi: 10.1016/j.jml.2008.11.002, PMID: 20126291 PMC2683024

[ref37] Staub A. (2010). Response time distributional evidence for distinct varieties of number attraction. Cognition 114, 447–454. doi: 10.1016/j.cognition.2009.11.003, PMID: 20003964

[ref38] Stump G. (2001). Inflectional Morphology. Cambridge: Cambridge University Press.

[ref39] Tanner D. Nicol J. Brehm L. (2014). The time-course of feature interference in agreement comprehension: multiple mechanisms and asymmetrical attraction. J. Mem. Lang. 76, 195–215. doi: 10.1016/j.jml.2014.07.003, PMID: 25258471 PMC4170797

[ref40] Tucker M. A. Idrissi A. Almeida D. (2021). Attraction effects for verbal gender and number are similar but not identical: self-paced reading evidence from modern standard Arabic. Front. Psychol. 11:3774. doi: 10.3389/fpsyg.2020.586464 PMC7859339 33551906

[ref41] Vigliocco G. Butterworth B. Garrett M. (1996). Subject–verb agreement in Spanish and English: differences in the role of conceptual constraints. Cognition 61, 261–298. doi: 10.1016/S0010-0277(96)00713-5, PMID: 8990974

[ref42] Vigliocco G. Butterworth B. Semenza C. (1995). Constructing subject–verb agreement in speech: The role of semantic and morphological factors. J. Mem. Lang. 34, 186–215. doi: 10.1006/jmla.1995.1009

[ref43] Vigliocco G. Franck J. (1999). When sex and syntax go hand in hand: gender agreement in language production. J. Mem. Lang. 40, 455–478. doi: 10.1006/jmla.1998.2624

[ref44] Villata S. Franck J. (2020). Similarity-based interference in agreement comprehension and production: evidence from object agreement. J. Exp. Psychol. Learn. Mem. Cogn. 46, 170–188. 31033310 10.1037/xlm0000718

[ref45] Wagers M. W. Lau E. F. Phillips C. (2009). Agreement attraction in comprehension: representations and processes. J. Mem. Lang. 61, 206–237. doi: 10.1016/j.jml.2009.04.002

[ref46] Wiese B. (2004). “Categories and paradigms. On underspecification in Russian declension,” in Explorations in Nominal Inflection. eds. Müller G. Gunkel L. Zifonum G. (Berlin: Mouton De Gruyter), 321–372.

[ref47] Wunderlich D. (2004). “Is there any need for the concept of directional syncretism?” in Explorations in Nominal Inflection. eds. Müller G. Gunkel L. Zifonum G. (Berlin: Mouton De Gruyter), 373–395.

[ref48] Yanovich I. (2012). “What can russian gender tell about the semantics of phi-features?” in *Talk Given at the 21st Formal Approaches to Slavic Linguistics Conference*; May 11, 2012, United States.

[ref49] Zwicky A. (1991). “Systematic versus accidental phonological identity,” in Paradigms: The Economy of Inflection. ed. Plank F. (Berlin: Mouton de Gruyter), 113–132.

